# Practicability and environmental impact assessment of synthetic fibre reinforced polymer (SFRP) stirrups in reinforced concrete beams

**DOI:** 10.1016/j.heliyon.2024.e28149

**Published:** 2024-03-21

**Authors:** Balamurali Kanagaraj, Anand N, Samuvel raj R, Diana Andrushia, Eva Lubloy

**Affiliations:** a Department of Civil Engineering, Karunya Institute of Technology and Sciences, Coimbatore, India; b Department of Electronics and Communication Engineering, Karunya Institute of Technology and Sciences, Coimbatore, India; c Department of Construction Materials and Technologies, Faculty of Civil Engineering, Budapest University of Technology and Economics, Budapest, Hungary

**Keywords:** Environmental impact assessment, Flexure, Shear strength, Ductility, Sustainability

## Abstract

In this study, the objective is to explore the practicability of incorporating synthetic fibre reinforced polymer (SFRP) stirrups into reinforced concrete beams. This investigation revolves around evaluating their effectiveness from two key perspectives: their structural performance and environmental impact. To accomplish this, four set of specimens were prepared, each integrating SFRP stirrups, and testing them under a rigorous three-point bending load test. The structural performance analysis entails a comprehensive examination on the critical design factors such as: the load-deflection relationship and the contribution these SFRP stirrups to improve the ductility performance, flexural stiffness, deformability factor, flexural toughness and energy absorption capacity. The findings of this study indicate that the SFRP stirrups exhibit commendable shear capacity, meeting the necessary requirements, and simultaneously demonstrate satisfactory ductility. It is determined, that the optimal design for these SFRP stirrups involves utilizing narrow and thin stirrups placed at relatively larger intervals. Furthermore, this research delves into assessing the environmental impact of incorporating SFRP stirrups. This assessment enables us to comprehensively evaluate the environmental implications of the entire life cycle of these stirrups in structural beam. Moreover, the analysis reveals that, SFRP stirrups yields lower environmental impacts compared to their steel counterparts, they still provide valuable insights into the overall sustainability considerations within the context of reinforced concrete structures.

## 1 Introduction

The presence of pores and voids in the microstructure can impact the overall strength and durability of concrete; high porosity can lead to stress concentration points, making the material more susceptible to cracking and failure [1]. The process of cement hydration involves the chemical reaction between cement particles and water, forming hydrated products that contribute to the concrete's strength [2]. The rate and extent of hydration influence the development of the microstructure and, consequently, the stress distribution. The interaction between aggregates and the surrounding cement paste is critical; a strong interface enhances load transfer between the aggregates and the matrix, influencing how stresses are transmitted through the material [3].

The stress-strain relationship is generally related to physical characteristics as well as internal microcracking of materials, because of such a relationship, a stress-strain curve may be used as a means to determine indirectly the homogeneity of the material and some characteristics of the fracture [4]. The stress-strain curve of the cement paste is similarly linear to about 90% of its strength, indicating good homogeneity, whereas the stress-strain curve of the concrete, being inhomogeneous, deviates greatly from linearity at the stress less than 50% of the strength [5]. This deviation from the linearity has been related to the bond cracks between coarse aggregate and mortar matrix. The presence of these defects can alter the stress distribution within the concrete, leading to localized failures; up on loading, these bond cracks lead to globalized failure [6].

The presence of water and high quantities of chloride ions in maritime areas can cause significant corrosion of reinforcing steel in reinforced concrete (RC) buildings [7]. This corrosion phenomena are most noticeable in steel stirrups, which are placed nearest to the substrate's layer and thus exposed to the toughest environmental conditions. As a result, the steel stirrups are extremely subjected to corrosion [8]. The consequences of this corrosion are twofold. Firstly, it weakens the integrity and structural strength of the stirrups themselves, compromising their ability to withstand loads and contribute to the overall stability of the RC structure [9]. Secondly, the corrosion of steel stirrups has a direct impact on the shear capacity of the structure [10]. Shear capacity refers to the ability of a structural member to resist the transverse loads [11]. The corrosion-induced deterioration of the steel stirrups leads to a decrease in shear capacity, making the RC structure more susceptible to potential failure [12].

Additionally, the steel stirrups commonly used in RC structures are characterized by small diameters. This small diameter further exacerbates their susceptibility to localized corrosion, specifically known as pitting corrosion [13]. Pitting corrosion is a localized form of corrosion that causes the formation of small pits or holes on the surface of the stirrups. As these pits deepen and widen due to corrosion, they can significantly reduce the minimum required diameter of the stirrups [14]. Consequently, the diminished diameter of the stirrups further compromises their shear capacity, amplifying the structural vulnerability of the RC system. Therefore, it is essential to examine the soundness of structural element using non-destructive testing. Acoustic Emission (AE) is a non-destructive testing technique used for monitoring and detecting stress distribution, as well as the onset and progression of damage, in concrete structures [15]. It relies on the detection and analysis of transient stress-related acoustic signals produced by the material when subjected to external forces. When stress is applied to concrete, microstructural changes, crack initiation, or propagation can occur. These changes generate acoustic waves, or emissions, which are small stress-induced elastic waves; these waves were captured using sensors, converting them into electrical signals. The acquired signals are then analysed in terms of their amplitude, frequency, duration, and other characteristics. Different types of signals can indicate various phenomena, such as crack initiation, crack growth, or other stress-related events. Due to variation in signals localization can be spotted and this localization capability helps identify the specific location where stress occurred within the concrete structure [16]. Changes in the pattern or frequency of acoustic emissions can indicate evolving stress conditions, enabling early detection of potential structural issues. AE is particularly useful for detecting and monitoring the growth of cracks, which are indicative of stress concentrations.

Piezoelectric devices show outstanding performance in detecting stress distribution in concrete structures [17]. Piezoelectric materials generate an electric charge in response to mechanical stress, and this property is leveraged in sensors to monitor and measure stress-induced deformations in concrete. Piezoelectric materials, such as certain crystals or ceramics, exhibit the piezoelectric effect. This effect involves the generation of an electric charge in response to mechanical deformation or stress. Piezoelectric sensors, often in the form of patches or embedded elements, are strategically placed on or within the concrete structure. These sensors are designed to convert mechanical stress or strain into electrical signals. As stress is applied to the concrete structure, it undergoes deformation and strain. This deformation causes the piezoelectric material within the sensor to generate an electric charge proportional to the applied stress [18]. The generated electric charge is then converted into an electric signal, which are measured and analysed. The magnitude of the electric signal corresponds to the level of stress or strain in the concrete. By monitoring and analysing these signals, it is easy to quantify the stress distribution and identify potential areas of concern. Based on these non-destructive testing methods it is easy to evaluate the structural performance of the element without breaking it and further suitable retrofitting techniques can be employed to safeguard the structure, when the condition is worse.

Reinforced concrete beams, slabs, joints, and frames can be constructed in various shapes, such as rectangular, square, and circular. They could be reinforced with either E-glass-epoxy or carbon-epoxy. FRP composite was utilized as the external reinforcement for beam-column joints in conventional building construction in the late 1990s [19]. A common technique for using FRP in reinforced concrete construction is by wrapping it either at a particular section or an entire surface [[20], [21], [22]]. The steel jacket application technology was first used same as CFRP wrapping technique [23]. Concrete and reinforced concrete structures are wrapped with FRP as external reinforcement to increase structural behaviour and performance. Different FRP wrapping techniques, like hand layup [24] and carbon anchor [25], are available, these strengthening process of building structural components. Karayannis et al. (2018) [26] examined RC beams with FRP bars as tensile reinforcement. The combination of long flexural cracks and low elastic modulus of FRP bars caused fracture in the beam. Before cracking, the member's stiffness was high because the entire concrete cross-section was used. After cracking, only the concrete compressed part was effective. In contrast, the FRP tensile bars' contribution was relatively smaller because of the material's low modulus of elasticity. The enhanced anchoring conditions for FRP bars improved the bond behaviour between the bars and the concrete, enhancing the flexural behaviour. Due to the significantly greater modulus of elasticity of the steel bars compared to one of the FRP bars, the decreasing load was not observed for steel RC beams. Due to low axial stiffness, the cracking occurred more rapidly in the early stages of FRP beam than in steel-reinforced beams.

Murugan & Kumaran, (2019) [27] in their study five beams were casted with varying reinforcement ratios of 0.73% and 1.24% were used to cast two beams with sand-coated GFRP bars and two beams with grooved GFRP bars, respectively. Reinforcement ratio of 0.73% was used to investigate the differences between GFRP rods and conventional steel rods. The steel rods were attached to the stirrups with mild steel wires, while the GFRP rods were bonded with stirrups using nylon zip ties. The ultimate loads of sand-coated and grooved rod-reinforced beams were 34 kN and 38 kN, which were 15% and 5% lower compared to the steel-reinforced beam. When the reinforcement ratio of GFRP beams increased to 1.08%, the ultimate load is improved to 50 kN and 56 kN, representing increase of 25% and 40% more than the sand-coated and grooved rod-reinforced beams, respectively. Increasing the reinforcing ratio in GFRP-reinforced beams raised the beam's ultimate load. An increase in the reinforcing ratio resulted in a smaller ultimate deflection for grooved rod beams compared to sand-coated rod beams. Muciaccia et al. (2022) [19] The bond strength of FRP anchors was significantly affected by the construction technique quality and the dryness or impregnation of the dowel component of the FRP anchor. First, the binding strength of FRP anchors might be drastically diminished due to improper hole preparation, improper adhesive placement, or nonvertical anchor, known as fibres misalignment. Anchors from CFRP should have a cross-sectional area at least twice as large as the CFRP reinforcement sheet mounted. This problem had a significant impact on the retention and failure performance of the FRP sheet just before the FRP anchor failed. After that, to be functional, an FRP anchor should be at least 13 mm longer than the FRP's width, followed by 60-degree maximum angle. It was suggested that the anchors overlap by at least 10 mm by putting them next to one another. Finally, as a general rule, more anchors of smaller diameter were better than fewer anchors of bigger diameter.

Extensive attempts [28,29], have been made to replace conventional stirrups with synthetic stirrups, which offer exceptional resistance to corrosion with a high strength-to-density ratio. Although fibre reinforced polymer (FRP)s was first developed largely for structural rehabilitation, FRP composites have gained substantial consideration in recent years as construction materials for new structures [30]. It is worth mentioning that FRP stirrups have a more confining effect than steel stirrups [31], which leads to increased ductility in the core concrete and higher resilience to buckling of main rebar in RC columns [32]. This feature emphasizes the usefulness of FRP stirrups in improving the structural performance and longevity of RC sections.

Despite its advantages, conventional fibre reinforced polymer (FRP) shear reinforcement has several limitations that need to be addressed [33]. Firstly, the production process of FRP reinforcement, particularly carbon FRP, has significant environmental risks. To align with the increasing focus on environmental concerns and climate change, there is a growing interest in replacing synthetic FRP materials with more environmentally friendly alternatives. Second, despite its great tensile strength, synthetic FRP is prone to early failure owing to stress localization [34]. Bending protruded FRP bars to make FRP hoops or stirrups leads in fibre elongation at the outermost corners and fibre shortening at the innermost corners [35]. This elongation mismatch might cause FRP hoops to fail before they achieve their maximum tensile strength [36]. According to previous research, this early deformation might diminish the residual yield strength of non-metallic FRP by 40%–70%. Finally, the cost of FRP stirrups is a barrier to their broad use in the building sector [37]. Assuming that the tensile strength of synthetic FRP is fully used, the cost of delivering the same shear capacity contribution as steel stirrups is roughly 8 to 12 times greater. The expensive cost of FRP stirrups prevents their widespread application in construction projects [38].

Natural fibre reinforced polymer (NFRP) offers an alternative to synthetic fibre reinforced polymer, utilizing natural fibres impregnated with epoxy resin to create the composite material [39]. Among the various natural fibre options available, including flax, jute, and hemp [40], flax fibres have gained significant popularity in NFRP due to their satisfactory mechanical properties and cost-effectiveness. Despite having lower tensile strength compared to synthetic fibres, natural fibres provide higher cost efficiency, with the cost of achieving an equivalent tensile load being notably lower, ranging from 20% to 50% compared to glass fibres [41]. In terms of flexural strengthening, using flax FRP to achieve a unit increase in load capacity proves to be approximately 20% more cost-effective than synthetic FRP [42]. This advantage stems from the widespread availability of natural fibres globally, with flax fibres being one-tenth the cost of carbon fibres [43]. Additionally, the elastic modulus of flax fibres closely matches that of concrete, promoting better composite deformation between flax FRP and concrete, thus minimizing common debonding failures and enabling the maximum utilization of flax fibber's tensile strength. Furthermore, natural FRP exhibits lower environmental impacts [44]. Natural FRP has showed potential as a feasible alternative for synthetic FRP in non-structural applications such as circuit boards and wall panels [45], greatly lowering energy consumption and carbon dioxide emissions. However, according to the authors' recent cradle-to-gate research [46], when natural FRP is utilized for structural strengthening, the environmental benefits become less substantial due to the excessive usage of epoxy resin and the relatively lower strength of natural fibres. As a result, more study is required to investigate the performance of natural FRP in structural components and optimize its environmental advantages [47].

In consideration of the well-established pros and cons of FRP, the primary objective of this study is to assess the feasibility of utilizing SFRP stirrups in RC beams. The investigation encompasses an evaluation of both the structural performance and environmental implications associated with the incorporation of SFRP stirrups. For this purpose, four set beams were meticulously manufactured and subjected to rigorous three-point bending tests. The SFRP stirrups were tied along the longitudinal reinforcement within the stage cage. The test findings unequivocally demonstrate a substantial improvement in the ductility index and energy absorption capacity of the reinforced geopolymer concrete (GPC) beams due to the utilization of SFRP stirrups. Additionally, an in-depth environmental impact assessment is conducted, comparing the performance of steel stirrups to that of SFRP stirrups. Notably, the evaluation reveals that SFRP stirrups result in lower environmental impacts compared to their steel counterparts.

## 2 Materials and methods

### 2.1 Materials

Low Calcium Fly Ash (FA) and Ground Granulated Blast Furnace Slag (GGBS) were employed as the precursor material for the concrete production [48]. Both the precursor materials were procured from the local dealer in Coimbatore city. Locally available Manufactured-Sand (M-sand) and crushed granite stones as coarse aggregate (CA) were employed as the filler material in the production of concrete. The specific gravity & moisture content of fine and coarse aggregates are 2.75, 1.37%, and 2.81 and 0.97%, respectively. Similar to precursor and filler materials, the alkaline solutions (sodium hydroxide and sodium silicate) were also procured from the local vendor in Coimbatore. Polycarboxylate ether-based superplasticizer (SP) were employed at a dose of 2%. The mix design employed in the present investigation is illustrated in Table 1.

**Table 1:** Mix design.

Binder (kg/m^3^)		Filler (kg/m^3^)		Activator (kg/m^3^)		
Fly Ash	GGBS	M-Sand	CA	NaOH	Na_2_SiO_3_	W/B ratio
245	105	864	1174	59.52	89.28	0.4

**Fig. 1:**
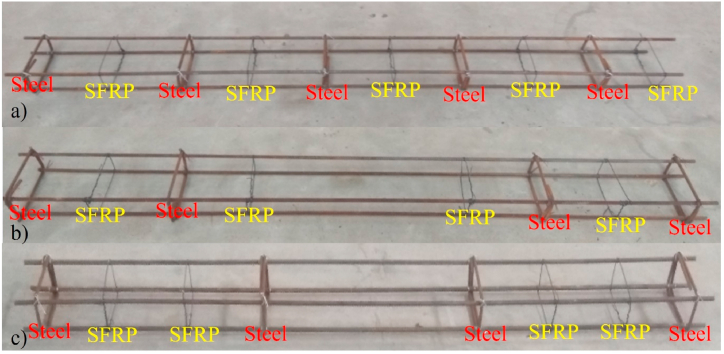
Placement of SFRP; a) alternate replacement of steel and SFRP (Beam-2); b) alternate replacement of steel with SFRP in shear zone (Beam-3); c) replacement of steel with SFRP in shear zone (Beam-4).

**Fig. 2:**
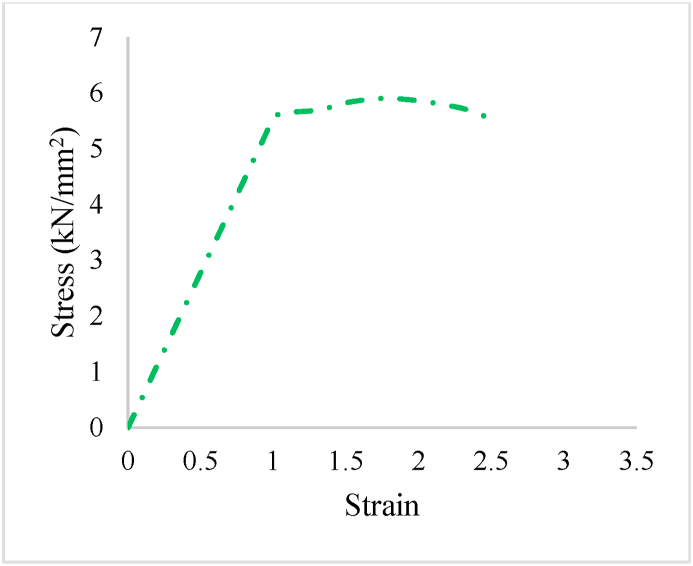
Stress-Strain behaviour of SFRP.

The present study examines the SFRP RC beams, with a dimension of 200 (B) × 200 (D) × 2000 (L) mm (Ref: Fig. 1). 4-# of 10 mm Ø bars were employed as longitudinal reinforcements for all the beams. 8 mm Ø bars were employed as stirrups with a center-to-center distance of 200 mm. Fig. 1a, shows the beam reinforcement with alternate replacement of steel and SFRP. Fig. 1b, shows the beam reinforcement with alternate replacement of steel with SFRP in shear zone. Fig. 1c, shows the beam reinforcement with replacement of steel with SFRP in shear zone. Comprehensive information regarding the reinforcement details of each specimen can be found in Table 2. Fig. 2, shows the stress-strain behaviour of SFRP. The maximum yield stress of the SFRP was found to be the range between 5.2 and 5.9 kN/mm^2^, with a maximum strain in the range between 2 and 2.5.

**Table 2:** Beam Specimen description.

Sample ID	Steel rebar		Steel Stirrups			SFRP Stirrups	
	Ø (mm)	No.	Ø (mm)	Spacing (mm)	No.	Spacing (mm)	No.
Beam-1	10	4	8	200	10	–	–
Beam-2	10	4	8	200	5	200	5
Beam-3	10	4	8	200	4	200	4
Beam-4	10	4	8	200	4	200	4

Key: Beam-1-Control specimen.

### 2.2 Experimental test program

**Fig. 3:**
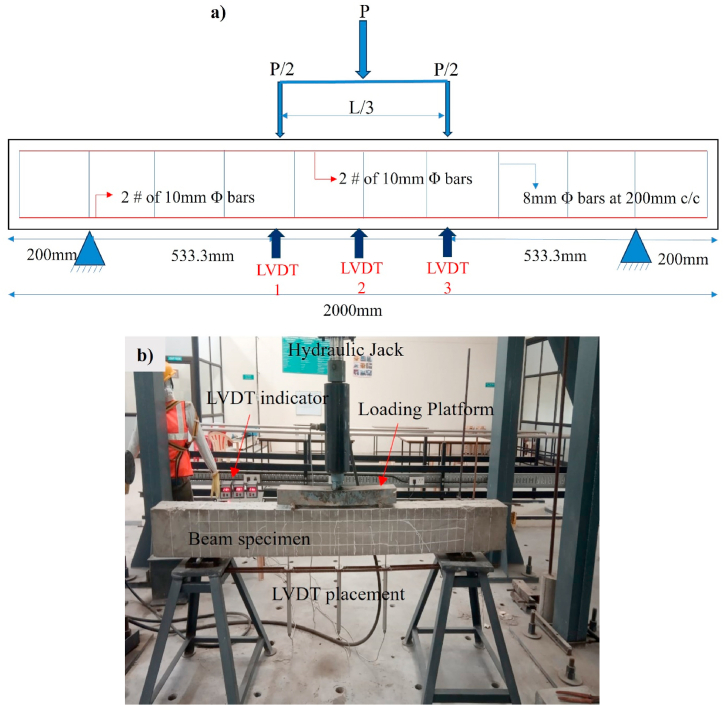
Test setup: a) Schematic representation; b) Experimental test setup.

To ensure accurate measurements and monitoring of the specimens during testing, three linear variable displacement transducers (LVDTs) were installed in each specimen. These LVDTs were strategically positioned at the 1/3-span and midspan locations, as shown in Fig. 3a. Their main purpose was to track and record any displacement that occurred during the testing process. To apply the load, a hydraulic loading frame with a capacity of 200T was utilized, as shown in Fig. 3. The loading rate was carefully set at 0.2 mm/min to maintain controlled and consistent testing conditions. To collect and record the gathered data effectively, all LVDTs were connected to a data acquisition system, ensuring precise and reliable sampling of the measurements obtained throughout the experiments.

### 2.3 Environmental impact assessment (EIA)

In this section, an evaluation of the environmental consequences related to the utilization of SFRP and steel stirrups were undertaken. The assessment centers on the concept of the "functional unit" (FU), enabling a comparison of the environmental effects stemming from different stirrup materials [49]. Through the examination of the growing utilization of both steel and SFRP stirrups, our objective is to provide a comprehensive analysis of their environmental implications.

The life cycle assessment (LCA) method, notably the cradle-to-gate approach, was used to ensure an equal and accurate evaluation. This sort of LCA considers the stirrups' partial life cycle, beginning with raw material extraction (cradle) and finishing with the manufacturing process (factory gate) [50]. This methodology is chosen due to the limited environmental impact of the stirrups during their use phase and the difficulties associated with quantifying the environmental ramifications of recycling or incineration of the stirrups following the demolition of RC structures. By scrutinizing the cradle-to-gate life cycle of both steel and SFRP stirrups, we can assess their respective environmental impacts and make comparisons regarding their sustainability performance. This evaluation will yield valuable insights into the environmental considerations associated with selecting stirrup materials for reinforced concrete structures.

The assessment of SFRP and steel stirrups involves a comprehensive life cycle inventory (LCI) analysis. For SFRP, the LCI boundary encompasses the entire life cycle, commencing from the manufacturing of high-density polyethylene (HDPE). On the other hand, the LCI boundary for steel stirrups begins with the excavation of steel ore and extends to the production of steel stirrups.

To assess the environmental impact of SFRP, the LCI of HDPE is considered, which involves multiple processes such as HDPE production, the market for HDPE plant, HDPE retting, and the market for HDPE products [51]. The assessment takes into account of various inputs, including chemicals and green materials used as raw materials in the production process. Moreover, the evaluation incorporates the consumption of treated water throughout the process, as well as the energy consumption related to steam and electrical power. These factors play a significant role in capturing the environmental impact associated with the production of HDPE, which is a vital component in the manufacturing of SFRP stirrups.

## 3 Results and discussions

### 3.1 Structural performance

#### 3.1.1 Load-deflection behaviour

**Fig. 4:**
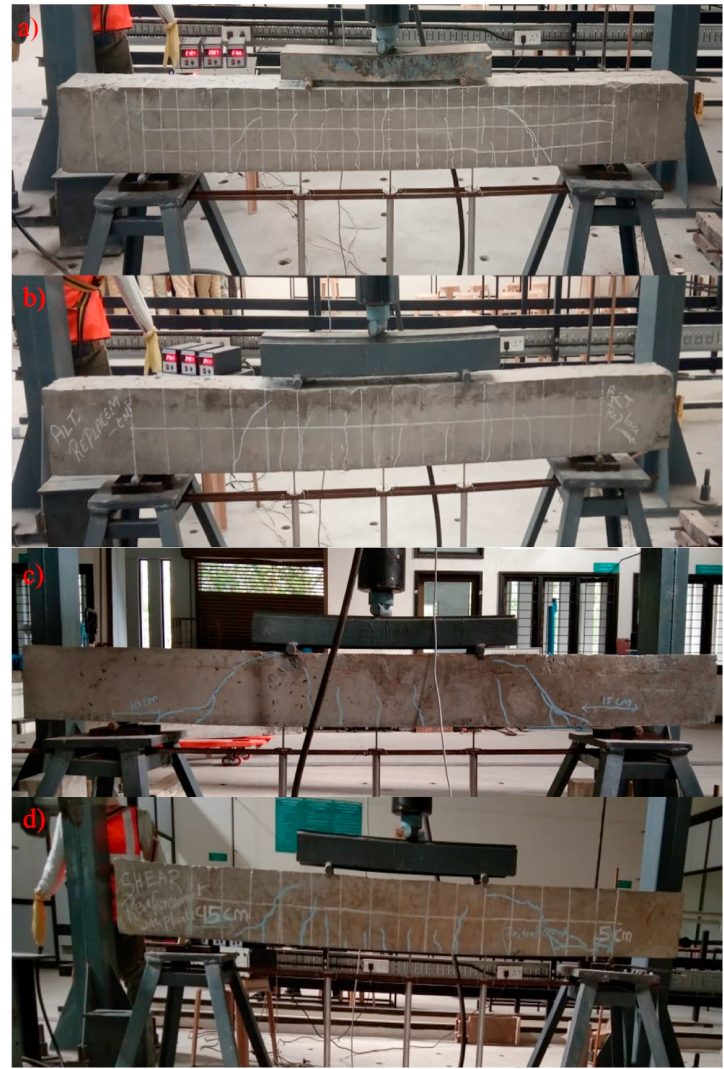
Failure pattern of beam specimens; a) Beam-1; b) Beam-2; c) Beam-3; d) Beam-4.

The primary objective of the experimental evaluation is to analyse and evaluate the flexural performance of GPC beams. The failure pattern of all four groups of GPC beams is presented in Fig. 4, highlighting their response under applied loads. The behaviour of these beams is influenced by the choice of embedded material employed across the cross-sectional area of the beams. Remarkably, even when SFRP is incorporated into the GPC beams, the slope of the load profile takes a linear path. This observation suggests that the inclusion of SFRP does not significantly alter the overall load-bearing characteristics of the GPC beams.

Additionally, the load-deflection response of the GPC beams follows a distinct pattern. Initially, the beams undergo a phase of tensile yielding, characterized by the development of cracks and the subsequent redistribution of stresses within the material. Following this stage, the beam reaches the yield threshold, indicating the point at which the material begins to exhibit significant plastic deformation. The control specimen exhibited flexural failure at a load of 52 kN. Similarly, the specimens incorporating steel and SFRP stirrups also experienced flexural failure, with their load-carrying capacities typically falling within the range of 51–58 kN. It is worth noting that flexural (Beam-1 (shown in Fig. 4a) & Beam-2 (shown in Fig. 4b)) and shear (Beam-3 (shown in Fig. 4c) & Beam-4 (shown in Fig. 4d)) failures were noticed in all the beam cases as depicted in Fig. 4(a–d).

The load required to induce the first fracture in the GPC beam offers valuable insights into the influence of SFRP on its behaviour. The experimental findings suggest that substituting the traditional steel stirrup with SFRP has a negligible impact on the load at the initial crack. This implies that the incorporation of SFRP does not significantly alter the initiation of cracking in the GPC beam. Throughout the testing process, micro-cracks were observed on the surface of the GPC specimen. These micro-cracks can be attributed to the typical shrinkage effect commonly associated with concrete materials. It is important to note that as referenced beams undergo loading, the load at the first fracture typically diminishes due to the loosening of the bond between the binder, such as the geopolymer matrix, and the steel reinforcement. This phenomenon may contribute to the observed formation of small-scale micro-cracks and the subsequent reduction in the load at the point of initial fracture.

**Fig. 5:**
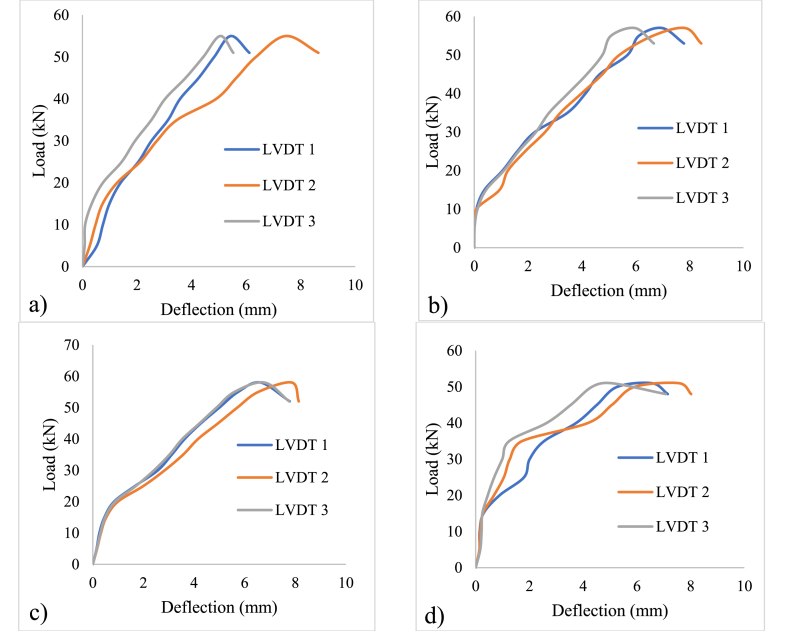
Load-Deflection pattern of beam specimens; a) Beam-1; b) Beam-2; c) Beam-3; d) Beam-4.

Fig. 5 illustrates the load-deflection relationship specific to the beam specimen. The plot demonstrates the specimen's behaviour under varying loads, indicating how the load increases in conjunction with deflection. This relationship provides valuable insights into the structural response and deformation characteristics of the specimen throughout the testing process. The beam specimens equipped with conventional steel stirrups (Beam-1) reached a peak load of 55 kN with a deflection of 7.49 mm (deflection at center of the beam) as shown in Fig. 5a. On the other hand, Beam-2 (as shown in Fig. 5b), Beam-3 (as shown in Fig. 5c), and Beam-4 (as shown in Fig. 5d) exhibited a similar load-deflection behaviour, as illustrated in Fig. 5b-d. The maximum load achieved for Beam-2 was 57 kN, and it experienced a maximum deflection of 7.83 mm (deflection at center of the beam). Beam-3 reached a maximum load of 58 kN with a peak deflection of 7.86 mm (deflection at center of the beam), while Beam-4 demonstrated a maximum load and deflection of 51 kN and 8.02 mm (deflection at center of the beam), respectively. Beam-1 and Beam-2, exhibited the flexural failure, whereas Beam-3 and Beam-4 have shown shear failure.

The load-deflection response of the SFRP stirrup, as illustrated in Fig. 5b-d, demonstrates a unique trend, which is different from the beam specimen with conventional steel stirrups. The interaction between the steel rebar and concrete matrix relies on the interlock facilitated by the ribbed surface of the reinforcement. When the concrete experiences flexural cracking, the mechanical interlock is compromised, resulting in a reduction in the load-carrying capacity. However, even in the presence of damage to the concrete substrate caused by flexural cracks, the SFRP stirrup maintains its composite action with the concrete substrate. This ensures that the width of the flexural crack remains controlled until the SFRP stirrup ruptures. Consequently, the deflection continues to increase, as indicated in Fig. 5. This behaviour is a result of the widening of the flexural crack and effectively prevents the commonly encountered issue of interfacial debonding that might be typically associated with SFRP materials.

#### 3.1.2 Shear capacity

A reinforced concrete beam's shear capacity describes how well it can withstand shear stresses. When shear forces surpass a beam's shear capacity, they operate perpendicular to the longitudinal axis of the beam and can cause it to deform or collapse [52]. A reinforced concrete beam's shear capacity is influenced by a number of variables, including the geometry of the beam, the strength of the concrete, and the quantity and placement of longitudinal and shear reinforcement [53]. Two processes, diagonal tension and aggregate interlock, together define the shear capacity of a reinforced concrete beam [54].

When a shear force is applied to a reinforced concrete beam, it is resisted by three main elements: the concrete itself, conventional stirrups, and SFRP stirrups. Each of these components contributes to the overall shear capacity of the beam [55]. The shear capacity of the concrete component is determined by two factors: concrete compression and the interlocking action of filler materials [56]. The concrete resists shear forces through its compressive strength, which depends on factors such as the concrete mix, curing process, and aggregate properties [57]. Additionally, the presence of filler materials, such as coarse aggregates, can enhance the interlocking effect and increase the shear capacity of the concrete [58].

Conventional stirrups, typically made of steel reinforcement, play a crucial role in resisting shear forces. They are placed along the beam's perimeter in a vertical orientation and provide tensile strength to counteract the shear stress. Beam specimen 1 exhibited a shear capacity of 28 kN, while Beam-2 showed a slightly higher shear capacity of 29 kN. Similarly, Beam-3 demonstrated a shear capacity of 29 kN, and Beam-4 had the lowest value of 26 kN.

Interestingly, the analysis revealed that the beams with and without SFRP reinforcement exhibited similar shear capacities with only minor variations. It is worth noting that despite of experiencing shear failure, SFRP-reinforced Beam-3 and Beam-4 were able to maintain their residual load carrying capacity. This suggests that the inclusion of SFRP reinforcement enhances the structural integrity and post-failure performance of the beams, allowing them to sustain some level of load even after experiencing shear failure.

#### 3.1.3 Moment-curvature relationship

The correlation among the imposed bending moment and the resultant curvature of a reinforced concrete beam is known as the moment-curvature relationship [59]. It is a key idea in structural engineering and it is applied to comprehend how reinforced concrete beams behave when subjected to bending stresses [60]. The moment-curvature relation is often nonlinear in a reinforced concrete beam. This implies that the curvature of the beam rises as the bending force increases, but the pattern is not proportional [61]. The beam first experiences elastic deformation as the bending moment rises, and the connection between moment and curvature adheres to Hooke's Law [62]. The stiffness of concrete and the reinforcement control this linear connection.

The beam approaches its yield point while the bending moment keeps growing [63]. The connection between moment and curvature is no longer linear at this point. The reinforcement starts to give way and the concrete in the compression zone begins to crack. The beam enters into the plastic stage after it has passed the yield point. As the link between moment and curvature gets more curved, it becomes clear that bigger moment increases lead to curvier increases [64]. Where the beam undergoes large localized rotations and plastic deformation, plastic hinges arise. The moment reaches its highest value in the absolute limit state, and the beam may fail or collapse. This collapse may be the result of overly aggressive concrete crushing, fractured reinforcing steel, or a breakdown in the link between the concrete and reinforcement.

**Fig. 6:**
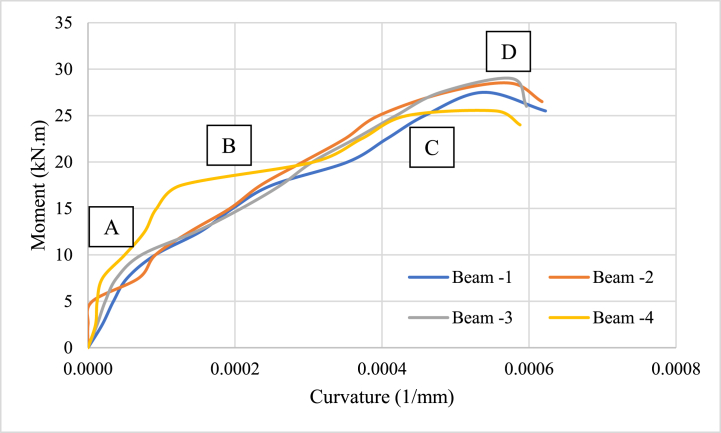
M-φ relation of SFRC reinforced concrete beams.

Fig. 6, shows the moment-curvature relationship of a reinforced concrete beam with four stages.

• Elastic Stage (A): In the initial stage, when the applied moment is relatively low, the beam behaves elastically, and the relationship between moment and curvature is linear. The beam deforms proportionally to the applied moment within its elastic range.
• Yielding Stage (B): As the moment increases, the tension reinforcement in the beam starts to yield (reaches its yield strength). The curvature also increases, but the relationship between moment and curvature becomes nonlinear. The beam begins to exhibit some plastic deformation, and the curvature increases at a faster rate.
• Post-Yielding Stage (C): In this stage, the tension reinforcement has yielded, and the beam continues to deform plastically. The moment-curvature relationship remains nonlinear, and the curvature increases rapidly with a small increase in moment. The beam undergoes significant plastic deformation, and the curvature increases even after the applied moment decreases.
• Ultimate Stage (D): The ultimate stage is reached when the beam reaches its maximum capacity and starts to fail. The curvature increases rapidly, and the moment decrease as cracks develop and propagate in the concrete. Eventually, the beam experiences a sudden drop in moment and loses its load-carrying capacity.

### 3.2 Performance index

#### 3.2.1 Ductility index

The ductility index is a measurement used to determine how ductile a material or structural component performs under bending [65]. A material's ductility, or capacity to absorb energy and undergo plastic deformation, is measured by how much substantial deformation or elongation it can withstand before failing [66]. For buildings to show warning indications and offer a margin of safety before total breakdown, this attribute is essential. The deflection or deformation of the material or structural element is compared at various stages of loading to determine the ductility index [67]. Equation (1) showcase that how ductility index can be determined. It is expected to compare the deflection at the first fracture (the beginning deformation) with the deflection at the ultimate load (the highest distortion before failure).

(1) $$\delta =\frac{\omega }{\Delta_{ℵ}}$$

Where, δ is the ductility index, ω is the ultimate load at which the specimen fails, $\Delta_{ℵ}$ is the deflection at which initial fracture occurred in the element.

**Fig. 7:**
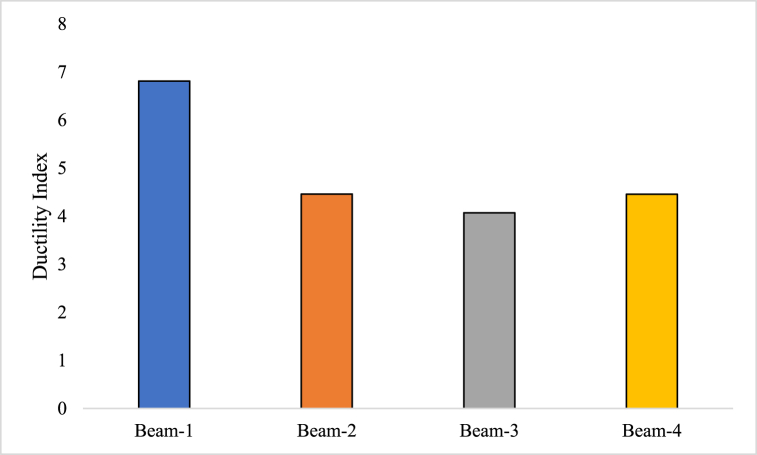
Ductility Index of GPC beam specimens.

Beam-1 demonstrates a ductility index of 6.81, indicating its ability to undergo deformation and absorb energy before failure. Similarly, Beam-2, Beam-3, and Beam-4 exhibit comparable ductility with slight variations. The ductility index for Beam-2 is found to be 4.46, while Beam-3 and Beam-4 have ductility indices of 4.07 and 4.46, respectively, as shown in Fig. 7. These values suggest that all beams possess a relatively high level of ductility, enabling them to sustain significant deformation and exhibit warning signs before ultimate failure occurs.

A material or structural component may be more ductile if it can sustain more deformations before failing, as indicated by a higher ductility index. In contrast, a lower ductility index denotes a material or structural component's constrained ability to deform as well as a more brittle behaviour. The ductility index is important in the context of structural design because it helps to assess the performance, dependability, and safety of materials and structures. It is frequently employed when evaluating and designing RC structures to make sure they have enough ductility to support applied loads and give early warning of impending failure.

#### 3.2.2 Flexural stiffness

The capacity of a structural element, such as a beam or member, to resist bending when subjected to an external load is referred to as flexural stiffness (Ƙ) [68]. It denotes the element's ability to resist deformation and preserve its shape in the face of bending moments [69]. Equation (2) showcase that how stiffness can be determined. A member's physical parameters, such as its moment of inertia and cross-sectional area, as well as the material's properties, such as its modulus of elasticity, affect the member's flexural stiffness [70]. A greater resistance to bending and a more rigid reaction to applied stresses are both indicated by a higher flexural stiffness.

(2) $$Ƙ=\frac{P}{\Delta }$$

Where, Ƙ represents the stiffness, P represents the load, and Δ represents the deflection of the beam specimens.

**Fig. 8:**
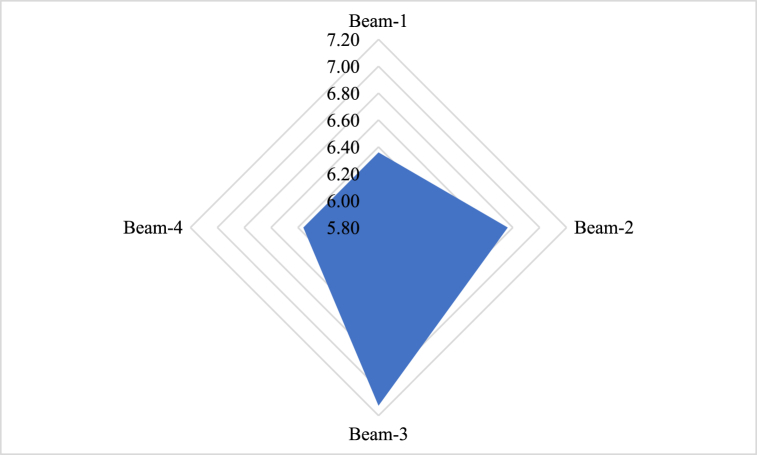
Flexural stiffness of GPC beam specimens.

The flexural stiffness of Beam-1 was determined to be 6.36 kN/mm, indicating a relatively higher stiffness compared to the other beams in the study. On the other hand, Beam-2, Beam-3, and Beam-4 exhibited similar flexural stiffness values with marginal fluctuations when compared to the reference beam (Beam-1). Specifically, Beam-2 displayed a flexural stiffness of 6.76 kN/mm, while Beam-3 and Beam-4 had flexural stiffness values of 7.13 kN/mm and 6.36 kN/mm, respectively, as shown in Fig. 8.

These results suggest that all the beams possess a significantly higher resistance to bending and deformation compared to the other beams. The flexural stiffness values observed in Beam-1, Beam-2, Beam-3, and Beam-4 indicate that these beams are relatively more flexible and prone to larger deflections under applied loads. The differences in flexural stiffness between the beams can be attributed to variations in their geometric properties, such as cross-sectional dimensions, and the material characteristics, including the modulus of elasticity.

It is important to consider the implications of flexural stiffness in structural design and analysis. Beams with higher flexural stiffness can provide enhanced structural stability, reduced deflections, and improved load-carrying capacity. On the other hand, beams with lower flexural stiffness may be suitable for applications where flexibility and controlled deflections are desired.

#### 3.2.3 Flexural toughness factor

When a material or structure is subjected to bending or flexural stresses, a metric known as the flexural toughness factor, also known as the flexural toughness index, is used to measure how well it can tolerate deformation and prevent fracture propagation [71]. It serves as a gauge of how resilient a material or structural component is under bending forces [72]. The area beneath the load-deflection curve produced from a flexural test done on the material or structure is compared in the computation of the flexural toughness factor [73]. Equation (3) showcase that how flexural toughness factor can be determined. This behaviour reflects the capacity of material or structure for its energy absorption as well as its resistance to the spread of fractures.

(3) $$\sigma_{b}=\frac{Tb}{\delta_{tb}}\frac{l}{{bh}^{2}}$$

Where, $\sigma_{b}$ is flexural toughness factor, T_b_ is flexural toughness, $\delta_{tb}$ is deflection of 1/150 of span

**Fig. 9:**
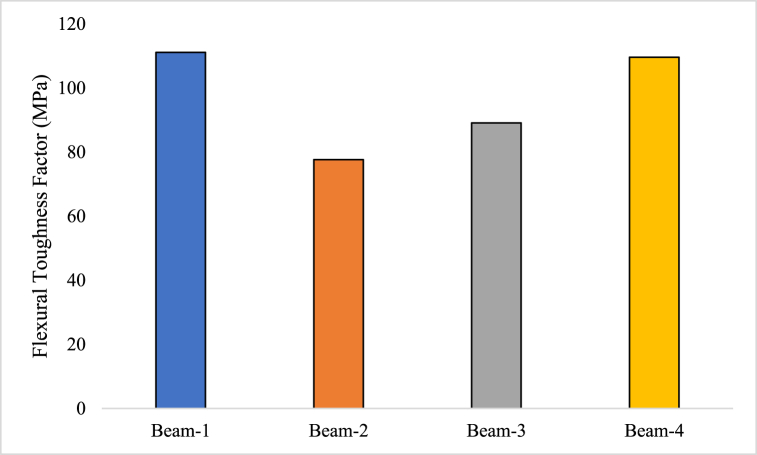
Flexural Toughness Factor of GPC beam specimens.

Beam-1 has a flexural toughness factor of 111.11 MPa. This means that Beam-1 can absorb a significant amount of energy and is highly resistant to fracture or failure under bending loads. It indicates that Beam-1 has a high capacity to withstand applied forces without breaking. Beam-2, on the other hand, has a flexural toughness factor of 77.63 MPa. This value suggests that Beam-2 is relatively less able to absorb energy and it is somewhat more susceptible to fracture or failure compared to Beam-1. Beam-3 has a higher flexural toughness factor of 89.10 MPa, indicating that it has better energy absorption and resistance to fracture compared to Beam-2 but it is still lower than Beam-1. Beam-4 has a flexural toughness factor of 109.60 MPa, which is closer to Beam-1, as shown in Fig. 9. This suggests that Beam-4 has a high capacity to absorb energy and resist fracture under bending loads, similar to Beam-1.

#### 3.2.4 Deformability factor

A measurement used to evaluate a material or structure's ability to deform when subjected to external stresses is the deformability factor, also known as the deformability index [74]. It gauges the relative amount of deformation or displacement that a material or structure exhibits when forces are applied [75]. The deformability factor of the RC beam was estimated using equations (5), (6). Comparing the actual deformation or displacement that has been observed in a material or structure with the anticipated or predicted deformation that has been estimated through theoretical models or design calculations is normally how the deformability factor is calculated [76]. This comparison sheds light on how well the material or structure conforms to the anticipated behaviour under particular loading circumstances.

(4) Deformabilityfactor=Strengthfactor×Deflectionfactor

(5) $$\text{Strength}\;\text{factor}=\frac{\omega }{\omega_{ℶ}}$$

(6) $$\text{Deflection}\;\text{factor}=\frac{\Delta }{\Delta_{ℶ}}$$

Where, ω is the ultimate load at which the specimen fails, $\omega_{ℶ}$ load at which 0.001 strain occurred in concrete, Δ is the ultimate deflection, $\Delta_{ℶ}$ deflection at which 0.001 strain occurred in concrete.

**Fig. 10:**
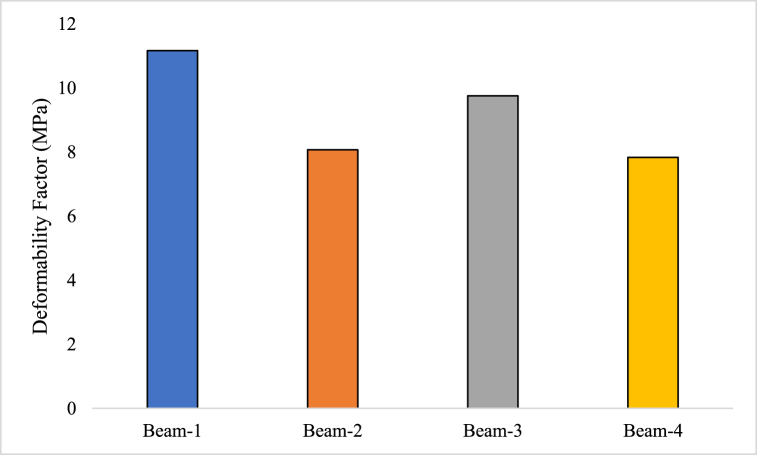
Deformability Factor of GPC beam specimens.

The deformability factor of different beams (Beam-1, Beam-2, Beam-3, and Beam-4) is being discussed. The deformability factor is a measure of how much a material or structure can deform or bend under a given load or stress. It indicates the ability of the beam to withstand and absorb external forces without breaking or undergoing excessive deformation. Beam-1 has a deformability factor of 11.6 MPa. This means that when subjected to a given load or stress, Beam-1 can deform up to 11.6 mm per unit of pressure (megapascal) applied. The higher deformability factor suggests that Beam-1 is more flexible or bendable compared to the other beams mentioned. Beam-2 has a lower deformability factor of 8.07 MPa, indicating that it is less flexible or more rigid compared to Beam-1. Similarly, Beam-3 and Beam-4 have deformability factors of 9.75 and 7.83 MPa, respectively, suggesting that Beam-3 is more flexible than Beam-2 but less flexible than Beam-1, while Beam-4 is the least flexible among the mentioned beams, as illustrated in Fig. 10.

#### 3.2.5 Energy absorption capacity

When subjected to external pressures or loads, a material or structure's ability to absorb and disperse energy is referred to as its energy absorption capacity [77]. It determines the maximum amount of energy that a material or building can hold before failing [73]. The ability of a structure to withstand and disperse energy during events like sudden impacts, earthquakes, or dynamic loads is indicated by a property known as energy absorption capacity, which is crucial in the area of structural engineering design problems [78]. A building with a larger energy absorption capacity has more efficiently absorb and release energy, which lowers the chance of damage or failure [65]. The strength, toughness, ductility, and deformation capacity of a material or structure are some of the elements that affect its ability to absorb energy [37]. Stronger and more resilient materials often have better energy absorption capabilities because they can sustain heavier loads and more deformations without breaking [61]. In order to construct durable and safe structures, it is crucial to understand how much energy a material or structure can absorb [79].

(7) $$\text{Energy}\;\text{Absorption}\;\text{capacity}=\int_{0}^{D}F\left(x\right)dx$$

Where, F(x) is the load (force) at a given deflection x, D is the maximum deflection of the beam.

**Fig. 11:**
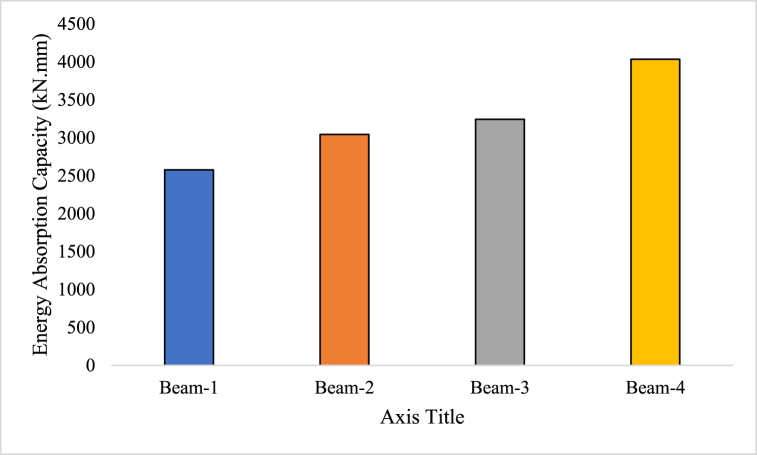
Energy Absorption Capacity of GPC beam specimens.

Equation (7) shows the formula to calculate the energy absorption capacity of the RC beam. The energy absorption capacity of Beam-1 was determined to be 2576.67 kNmm. In contrast, Beam-2, -3, and -4 exhibited higher energy absorption capacities in comparison to the reference beam (Beam-1). Specifically, Beam-2 had an energy absorption capacity of 3043.33 kNmm, while Beam-3 and Beam-4 had capacities of 3243.33 kNmm and 4033.33 kNmm, respectively, as shown in Fig. 11. This suggests that Beam-2, -3, and -4 have a greater ability to absorb and dissipate energy compared to Beam-1. The higher energy absorption capacities of these beams indicate their enhanced resilience and ability to withstand external forces or loads, such as impacts or dynamic loads. This characteristic is vital for ensuring the structural integrity and safety of the beams under various loading conditions. The increased energy absorption capacities observed in Beam-2, -3, and -4 can be attributed by the influence of SFRP. These beams have demonstrated a greater capability to absorb and dissipate energy, which is advantageous in applications where impact resistance and energy dissipation are critical, such as in seismic or high-velocity loading scenarios.

### 3.3 Environmental impact assessment

#### 3.3.1 Cost-efficiency

The capacity to achieve desired goals or objectives while reducing expenses and maximizing the value realized from the resources spent is referred to as cost-efficiency [80]. It entails determining the work or project's most efficient and cost-effective method of completion. Cost-efficiency in project management focuses on attaining objectives and delivering necessary outputs while avoiding costs, waste, and inefficiencies [81]. It comprises evaluating many options, approaches, and solutions to determine the most economical strategy that combines price, performance, and quality [82].

Cost-efficiency takes into account things like resource costs, material costs, labour costs, equipment costs, and other project-related costs [83]. The objective is to produce desired results while staying within the parameters of the budget or available resources, guaranteeing optimal resource use and maximizing return on investment [84]. Cost-cutting, process simplification, increased output, waste elimination, resource allocation optimization, and educated decision-making are frequently involved in achieving cost-efficiency [85]. To find areas for improvement and implement cost-effective solutions, thorough planning, monitoring, and assessment are necessary [86].

(8) $$\text{Cost}-\text{efficiency}=\frac{ultimate\;load\;bearing\;capacity}{cost\;of\;production\;of\;RC\;beam}$$

Equation (8), shows the cost-efficiency assessment of RC beam. The cost-efficiency analysis presented in Table 3 reveals the relative cost-effectiveness of different beams. Based on the data provided, Beam 1 demonstrates a cost-efficiency of 5.74 kN/$/beam, which means that for every dollar invested, it yields a load output of 5.74 kN. Similarly, Beam 2 shows a cost-efficiency of 6.84 kN/$/beam, indicating a slightly higher load output for the same investment. Beam 3, on the other hand, exhibits a cost-efficiency of 6.26 kN/$/beam, suggesting a moderate force output per dollar invested. Lastly, Beam 4 showcases the highest cost-efficiency among the four beams, with a value of 7.12 kN/$/beam, indicating that it provides the most force output for the investment made.

**Table 3:** Cost-efficiency analysis.

Sl.no	Materials	Rate	SFRC-Beam 1		SFRC-Beam 2		SFRC-Beam 3		SFRC-Beam 4	
		($/MT)	Qty (kg)	Cost ($)	Qty (kg)	Cost ($)	Qty (kg)	Cost ($)	Qty (kg)	Cost ($)
1	FA	13.05	11.03	0.14	11.03	0.14	11.03	0.14	11.03	0.14
2	GGBS	67.88	4.73	0.32	4.73	0.32	4.73	0.32	4.73	0.32
3	CA	24.14	52.83	1.28	52.83	1.28	52.83	1.28	52.83	1.28
4	MS	26.55	38.88	1.03	38.88	1.03	38.88	1.03	38.88	1.03
5	SS	117.48	2.68	0.31	2.68	0.31	2.68	0.31	2.68	0.31
6	SH	411.18	4.02	1.65	4.02	1.65	4.02	1.65	4.02	1.65
7	Steel	788.25	5.48	4.32	4.53	3.57	4.31	3.40	4.31	3.30
8	SFRC	121.27	–	–	0.15	0.0182	0.12	0.0146	0.12	0.0146
										
	Total Cost ($/beam)			9.06		8.33		8.15		8.15
										
	Ultimate Load (kN)			52		57		51		58
										
	Cost-efficiency (kN/$/beam)			5.74		6.84		6.26		7.12

Note: MS- M-Sand; SS- Sodium Silicate; SH- Sodium Hydroxide.

#### 3.3.2 Energy-efficiency

Energy efficiency is the ability to carry out tasks with the least amount of energy use or waste. It entails making effective and efficient use of energy resources in order to achieve the required level of productivity, service, or comfort [87]. Energy efficiency in the context of energy use focuses on maximizing the advantages or output from each unit of energy input in order to optimize energy use and reduce energy waste [88]. The objective is to use less energy while maintaining or improving performance, functionality, or comfort.

Transportation, commercial, industrial, and residential sectors have the benefits from energy efficiency [89]. It comprises of putting policies in place and utilizing technology that reduce energy losses, improve energy conversion procedures, and promote wise use of energy resources [90]. Utilizing energy-efficient appliances and equipment, applying insulation and weatherization techniques, improving heating, ventilation, and air conditioning systems, implementing efficient lighting technologies, and putting intelligent energy management systems in place are a few examples of energy efficiency practices [91].

(9) Energyefficiency=ΣEnergyindexfactor×ΣOverallmaterialsrequiredfortheproductionofconcrete

Energy efficiency of RC beams was estimated based on equation (9) and it has several benefits. It contributes to energy cost reduction, greenhouse gas emission reduction, resource conservation, increased energy security, and sustainable development [92]. Energy-efficient techniques decrease the total environmental effect connected with energy production and consumption, which in turn helps to promote a healthy environment [93]. Energy efficiency should be prioritized by governments, businesses, and people in order to prevent climate change, improve energy sustainability, and reap long-term economic and environmental advantages.

**Fig. 12:**
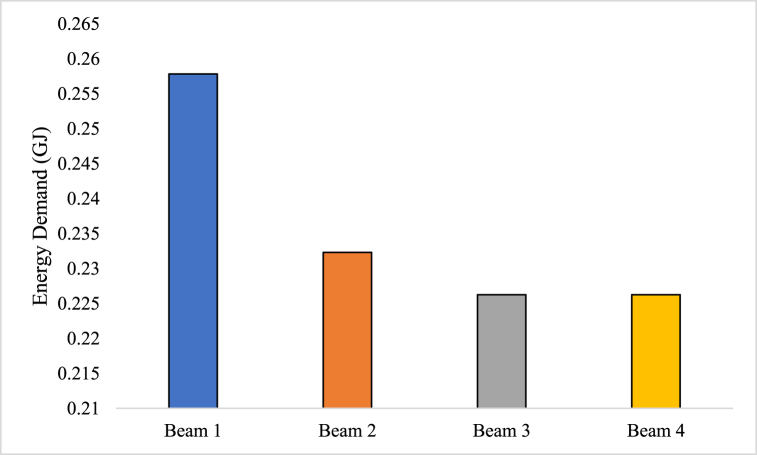
Energy-efficiency analysis.

The energy efficiency analysis depicted in Fig. 12 provides insights into the energy requirements for the production of different beams. According to the information provided, the following energy consumption values were observed: Beam 1: The production of Beam 1 necessitated an energy input of 0.2578 GJ (GJ). This means that 0.2578 GJ of energy were expended during its manufacturing process. Beam 2: Beam 2, on the other hand, required a slightly lower energy input of 0.2323 GJ. This implies that the production of Beam 2 consumed 0.2323 GJ of energy. Beam 3 and Beam 4: Both Beam 3 and Beam 4 exhibited the same energy requirements, with each of them demanding 0.2262 GJ of energy. This indicates that the manufacturing processes for these beams consumed 0.2262 GJ of energy.

#### 3.3.3 Carbon-efficiency

Carbon efficiency is the ability to carry out operations with the least amount of carbon dioxide (CO_2_) released into the atmosphere [94]. It entails lowering the carbon footprint connected to the creation, maintenance, or utilization of a good, service, or system [95]. Carbon efficiency is concerned with maximizing resource usage and decreasing carbon emissions across diverse activities, industries, or sectors with regard to sustainability and environmental effect. It entails implementing behaviour, innovations, and plans geared on reducing greenhouse gas emissions, with a focus on CO_2_, which is the main cause of climate change [96]. Carbon efficiency applies to a variety of human endeavours, including energy generation, travel, production, building, and daily living [97]. It comprises using sustainable practices, renewable energy sources, and energy-efficient technology to reduce carbon emissions.

The goal of carbon efficiency is to provide necessary services or achieve desired results while reducing carbon footprint [98]. Enhancing energy efficiency, encouraging the use of renewable energy, applying cleaner production techniques, adopting sustainable transportation options, and taking part in carbon offset or mitigation activities will be helpful to achieve this [99]. By placing a high priority on carbon efficiency, individuals, enterprises, and governments can actively combat climate change, preserve natural resources, promote environmental sustainability, and create a resilient, low-carbon future [100,101].

(10) Carbonefficiency=ΣCarbonindexfactor×ΣOverallmaterialsrequiredfortheproductionofconcrete

**Fig. 13:**
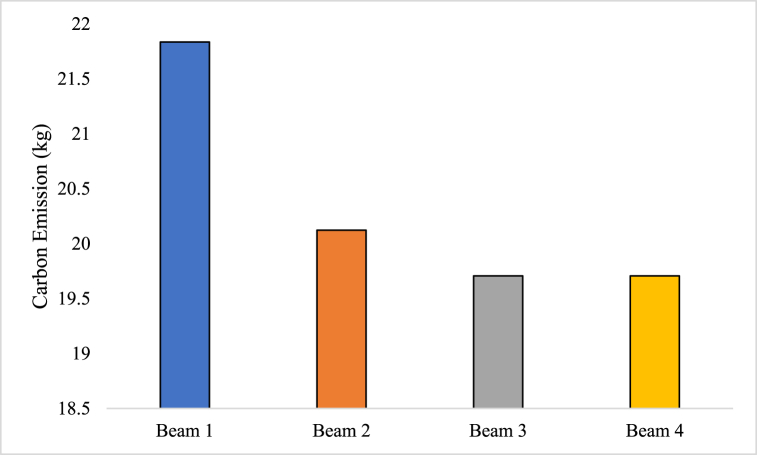
Carbon-efficiency analysis.

Energy efficiency of RC beams was estimated based on equation (10) and it has several benefits. Fig. 13 illustrates the carbon efficiency analysis conducted on the four beams. The analysis provides valuable information about the carbon emissions associated with the production of each beam. The results are as follows: Beam 1: During the production process, Beam 1 was found to generate 21.83 kg of CO_2_. This indicates that the manufacturing of Beam 1 resulted in the release of 21.83 kg of CO_2_ into the atmosphere. Beam 2: The carbon emissions associated with Beam 2 were measured to be 20.12 kg of CO_2_. This implies that the production of Beam 2 contributed 20.12 kg of CO_2_ to the overall carbon footprint. Beam 3 and Beam 4: Both Beam 3 and Beam 4 exhibited the same carbon emission value of 19.70 kg. Therefore, the manufacturing processes for both beams resulted in the release of 19.70 kg of CO_2_.

## 4 Scope for futuristic studies

Based on the experimental investigation and sustainability analysis conducted, it is recommended that the utilization of SFRP stirrups should be optimized in more detail. Here, some of the key points are given to consider.

• Increasing Stirrup Thickness: To effectively handle higher shear stresses, it is advisable to increase the thickness of the SFRP stirrups. By doing so, the stirrups will have enhanced strength and capacity to withstand greater shear forces.
• Finite Element Analysis: Conducting further finite element analysis (FEA) is crucial to validate the findings of the experimental investigation. FEA can provide a more comprehensive understanding of the structural behaviour of SFRP stirrups and facilitate the development of a performance-based design approach. Also, parametric analysis will be helpful to achieve the optimum requirement of SFRP stirrups.
• Bond Performance and Element-Level Investigation: To showcase the performance of SFRP stirrups against conventional materials, it is essential to evaluate their bond performance. This involves investigating the interaction between SFRP stirrups and concrete at the element level, focusing on aspects such as load transfer and anchorage.
• Durability Assessment: To ensure the long-term viability of SFRP stirrups, a thorough investigation into their durability is necessary. This analysis should include assessing their resistance to environmental factors such as moisture, temperature variations, and chemical exposure, to ensure their effectiveness and longevity in real-world applications.
• Seismic performance analysis and behaviour of SFRP beams has to be studied in detail.
• Studies on influencing parameters affect the flexural performance of beam with SFRP using machine learning approach.

By addressing these aspects through further optimization, analysis, and testing, a more robust understanding of the performance, design considerations, and durability of SFRP stirrups can be obtained. This will facilitate their successful integration into construction practices, promoting sustainable and efficient structural solutions.

## 5 Conclusion

The objective of this study is to investigate the use of SFRP as an alternative to conventional steel stirrups for shear reinforcement in RC beams. The study compared the EIA and structural performance of beams with conventional stirrups and those with SFRP stirrups. Based on the experimental investigation conducted, the following key conclusions were drawn.

• The RC beams reinforced with SFRP stirrups exhibited similar performance to conventional beam specimens in terms of load-deflection behaviour. This indicates that SFRP stirrups can effectively provide the required shear reinforcement in RC beams without compromising their overall structural behaviour.
• All the RC beams, regardless of whether they had conventional stirrups or SFRP stirrups, demonstrated similar shear capacity under the applied loading conditions. This suggests that SFRP stirrups can effectively resist shear forces and perform comparably to conventional stirrups.
• In terms of performance indices, all the beams exhibited a relatively high level of ductility, energy absorption capacity, and flexural stiffness. This indicates that both the conventional and SFRP-strengthened beams have desirable structural properties and can effectively withstand and dissipate energy under load.
• From an environmental impact perspective, Beam 3 and Beam 4 demonstrated higher cost-efficiency, lower energy demand, and lower carbon emissions compared to Beam 1 and Beam 2. This suggests that the use of SFRP stirrups in RC beams can have positive environmental implications by reducing resource consumption and carbon emissions associated with conventional steel stirrups.

## Data availability

Data will be available based on the request.

## CRediT authorship contribution statement

**Balamurali Kanagaraj:** Writing – original draft. **Anand N:** Supervision, Methodology, Conceptualization. **Samuvel raj R:** Writing – review & editing, Investigation. **Diana Andrushia:** Writing – review & editing. **Eva Lubloy:** Writing – review & editing, Funding acquisition.

## Declaration of competing interest

The authors declare that they have no known competing financial interests or personal relationships that could have appeared to influence the work reported in this paper.
